# Reproductive Toxicity of Triptolide in Male House Rat, *Rattus rattus*

**DOI:** 10.1155/2014/879405

**Published:** 2014-10-13

**Authors:** Neena Singla, Swati Challana

**Affiliations:** Department of Zoology, Punjab Agricultural University, Ludhiana 141004, India

## Abstract

The aim of study was to investigate the toxic effect of triptolide fed in bait on reproduction of male house rat, *Rattus rattus*. Feeding of cereal based bait containing 0.2% triptolide to male *R. rattus* for 5 days in no-choice feeding test, leading to mean daily ingestion of 20.45 mg/kg bw of triptolide, was found effective in significantly (*P* ≤ 0.05) reducing sperm motility and viability in cauda epididymal fluid by 80.65 and 75.14%, respectively, from that of untreated rats. Pregnancy rates were decreased by 100% in untreated cyclic female rats paired with male rats treated with 0.2% triptolide. Present studies suggest the potential of 0.2% triptolide bait in regulating reproductive output of *R. rattus*.

## 1. Introduction

The house rat,* Rattus rattus *(Linnaeus), one of the most common commensal rodent pests worldwide [1], is the predominant pest species infesting and depredating poultry farms in India with highest annual productivity of 69.59 young/female/year reported for any Indian rodent species [2, 3]. Poultry farms provide a most favourable and stable habitat throughout the year for large populations of* R. rattus* [4] which causes severe economic losses by both direct damage to poultry production and indirect damage by spreading several diseases among the poultry birds and to poultry keepers themselves [3, 5–9].

After natural reduction or control with rodenticides and other methods, rodents rapidly rebuild up their population [10] by enhancing their reproduction. Repeated use of rodenticides may lead to several problems including bait shyness, resistance, and other nontarget toxicity hazards [11, 12]. Although poisons may be useful in the initial reduction of a high density population and, thereby, in reducing the immediate damage caused by them, fertility control could be used to maintain the population at lower level. Because of their low mammalian toxicity, cost effectiveness, and easily biodegradable nature, plant products possess the potential in pest management [13].

Triptolide is one of the major compounds, identified in* Tripterygium wilfordii*, a twining vine of the family Celastraceae, as most promising for causing antifertility effects [14–19]. For field scale use of an antifertility agent, it is required to be fed in bait form. In India, for the first time, Singla et al. [20] reported antifertility potential of 0.1% triptolide mixed in cereal based bait and fed for 14 days to male* R. rattus *under laboratory conditions. However, for field application, it is again very difficult to feed bait containing triptolide to rats for such a long duration of time. Present studies were therefore undertaken to determine the effective concentration of triptolide which when fed in bait for shorter duration of time can control reproductive output of male* R. rattus.*

## 2. Material and Methods

The present study was carried out in the Animal House Laboratory, Department of Zoology, Punjab Agricultural University (PAU), Ludhiana, India.

### 2.1. Collection and Maintenance of Animals

Male* R. rattus* were live trapped with multicatch rat traps from poultry farms in and around Ludhiana. In the laboratory, rats were kept individually in cages (36 × 23 × 23 cm) for acclimatization for 10–15 days before the commencement of experiment with food and water provided* ad libitum*. Food consisted of a loose mixture of cracked wheat, powdered sugar and groundnut oil (WSO bait) in ratio of 96 : 2 : 2. Proper hygienic conditions were maintained. Approval of the Institutional Animal Ethics Committee was obtained for the usage of animals.

### 2.2. Treatment

Triptolide (molecular weight 360.41) used in present studies was kindly supplied by Pidilite Industries Pvt. Ltd., New Delhi, India. Mature and healthy rats (*n* = 24; average body weight 159.4 ± 15.2 g) were divided into four groups of six rats each. Rats of groups II–IV were fed on WSO bait containing 0.1, 0.2, and 0.3% triptolide, respectively, for 5 days in no-choice feeding test, whereas the rats of group I kept as untreated were fed on WSO bait only. Treatment bait was prepared as per the method described by Singla et al. [20]. Water was provided* ad libitum.*

### 2.3. Bait Acceptance

Before and after the treatment, rats of all the groups were fed on WSO bait. The consumption of WSO bait for pre- and posttreatment periods and treatment bait during treatment period was recorded after every 24 h and the mean daily intake of bait (g/100 g body weight (bw)) was determined. Before weighing, the bait of all the treated and untreated rats was cleared of faecal pellets and dried. Based on the amount of treated bait consumed, the total and mean daily dose (mg/kg bw) of triptolide ingested by each group of rats were calculated. Rats were also observed for mortality. The percent acceptance of treated bait over WSO bait consumed by each group of rats during pretreatment period was determined as per the formula given as follows:

(1) $$\begin{array}{c} \frac{\text{Consumption}\;\text{of}\;\text{treatment}\;\text{bait}\;\text{during}\;\text{treatment}\;\text{period}}{\text{Consumption}\;\text{of}\;\text{WSO}\;\text{bait}\;\text{during}\;\text{pretreatment}\;\text{period}}\times 100. \end{array}$$

### 2.4. Effect on Reproductive Output

After 15 days of termination of treatment, the treated and untreated male rats were paired with healthy, untreated, and cyclic female rats in ratio 1 : 1. Before pairing, the vaginal fluid of all the untreated female rats was examined twice a day for two weeks to determine their cyclic nature. During pairing, food and water were provided* ad libitum*. Food consisted of cracked wheat, powdered sugar, groundnut oil, and milk powder in ratio 91 : 2 : 2 : 5. In addition, soaked gram seeds were also provided. Pairing was carried out in breeding pens. After 15 days of pairing, male rats were separated and female rats were observed for pregnancy and delivery of pups.

### 2.5. Antifertility Effects

Thirty days after the termination of triptolide treatment, male rats of all the groups were weighed, anaesthetized, and autopsied to record the antifertility effects of triptolide. Their reproductive organs such as testis, epididymis, seminal vesicles, and prostate gland were dissected out, cleared of fat tissue, and weighed (g/100 g bw). One of the cauda epididymis of each rat was incised and pressed to take out the cauda epididymal fluid. The effect of triptolide on sperm motility (%), sperm viability (%), sperm density (millions/mL), and sperm morphology (% abnormality) in the cauda epididymal fluid was determined as per the methods described by Salisbury et al. [21] and Singla and Garg [22]. To determine abnormality in sperm morphology, the numbers of normal and abnormal sperms from Geimsa stained smears of cauda epididymal fluid were counted per 100 sperms at 400x. Sperms with head tail separation, acrosomeless heads, knob shaped heads, straight heads, triangular heads, banana shaped heads, heads coiled over midpiece, and coiled tail were considered abnormal. Smears of cauda epididymal fluid of untreated and treated rats were fixed in 2.5% buffered glutaraldehyde solution for 2 hours, washed with buffer, again fixed in 2% osmium tetroxide for half an hour, washed in buffer, dehydrated in graded ascending series of alcohol, air dried, and sputter coated for scanning electron microscopic (SEM) imaging of sperms for abnormalities in sperm morphology. Percent decrease in values of sperm motility, viability, and density in cauda epididymal fluid of treated groups of rats from that of untreated group of rats was also calculated.

### 2.6. Statistical Analyses

All values were expressed as mean ± SD. Significance of differences was determined using one-way analysis of variance and Tukey's test. The statistical analyses were performed using Graph Pad Instat Version 3.0 for Windows (Graph Pad Software, San Diego, CA, USA, at http://www.graphpad.com). Critical differences were considered significant at *P* ≤ 0.05.

## 3. Results and Discussion

### 3.1. Bait Acceptance

**Figure 1:**
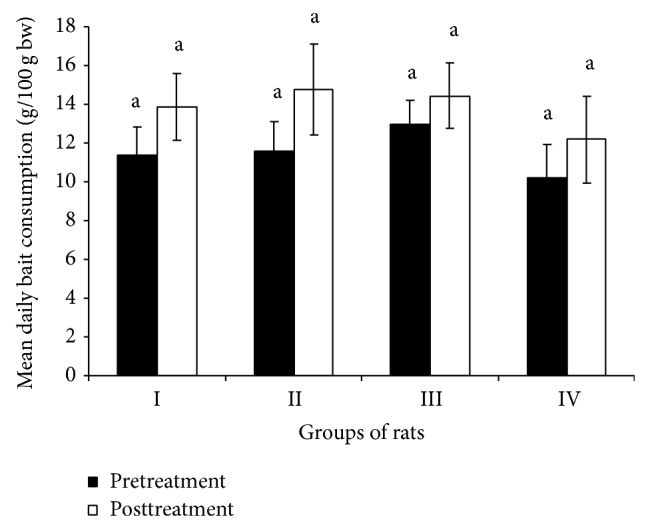
Mean daily consumption of WSO bait during pre- and posttreatment periods by different groups of rats.

Feeding of 0.1, 0.2, and 0.3% triptolide in bait for 5 days in no-choice feeding test revealed significantly (*P* ≤ 0.05) low consumption (g/100 g bw) of treated bait by rats of treated groups compared to the WSO bait consumed by rats of untreated group. The acceptance of treated bait over the plain bait consumed during pretreatment period by rats of groups II, III, and IV was found to be 93.0, 78.8, and 75.9%, respectively (Table 1). Feeding of treated bait to treated groups I, II, and III for 5 days in no-choice feeding test led to total ingestion of 53.6, 102.2, and 113.0 mg/kg bw of triptolide, respectively, with mean daily ingestion of 10.8, 20.4, and 22.6 mg/kg bw, respectively. Only one rat of group treated with 0.1% was found dead at the end of treatment. No mortality of rats was observed in other groups. There was no significant difference observed in consumption of WSO bait by different groups of rats between pre- and posttreatment periods (Figure 1) indicating no adverse effects of triptolide treatment on appetite of rats and hence subsequent bait consumption by rats after the treatment. Singla et al. [20] also did not report any adverse effects of triptolide treatment on posttreatment bait consumption. However, Liu et al. [23] reported anorexia, diarrhoea, leanness, and suppression of bait intake in male rats treated with triptolide at the dosages of 200 and 400 *μ*g/kg/day for 28 days.

### 3.2. Reproductive Performance

None of the untreated cyclic female rats (*n* = 3) paired with male rats fed on bait containing 0.2% triptolide for 5 days in no-choice feeding test delivered pups. However, one female out of the three paired with male rats fed on bait containing 0.1% triptolide delivered 8 pups while two females out of the three paired with male rats fed on bait containing 0.3% triptolide delivered 5 pups each. All the three female rats paired with untreated male rats were found positive for breeding as revealed by the presence of 5, 7, and 10 foetuses in their uteri after their sacrifice on day 15 after pairing (Table 2). Reduction in pregnancy rates during present studies was 66.67, 100, and 33.33% in rats treated with 0.1, 0.2, and 0.3% triptolide, respectively. No conception in female rats paired with male rats treated with 0.2% triptolide during present studies may be due to lowered values of sperm motility (10.00 ± 6.45%) and viability (14.50 ± 7.27%) observed in these rats compared to the values found in rats treated with 0.1 and 0.3% triptolide (Table 3).

Qian et al. [24] reported infertility with a drastic decrease in density and viability of epididymal spermatozoa in male rats treated with multiglycosides of* T. wilfordii* (GTW) at the dosage of 10 mg/kg/day for 8 weeks via gastric gavage. Miao et al. [25] carried out studies with GTW on farmland rats and mice at the dosage of 30 and 50 mg/kg/day and reported a decrease in birth rate by 32.6%. The pregnancy rates measured by housing each male with two untreated females were 100, 67, and 0% in control, low dose (50 *μ*g/kg bw/day), and high dose (100 *μ*g/kg bw/day) treated rats, respectively [15]. Huynh et al. [17] by housing each male with two untreated females measured 100 and 0% pregnancy rates in control rats and rats fed daily with 100 *μ*g/kg bw of triptolide for 82 days.

### 3.3. Antifertility Effects

Autopsy of all the male rats after 30 days of termination of treatment revealed no significant effect of triptolide treatment on weights (g/100 g bw) of testis, epididymis, seminal vesicles, and prostate gland (Table 3). Lue et al. [15] also did not observe any significant differences in mean weights of testis, epididymis, ventral prostate, and seminal vesicles among untreated rats and rats administered 50 and 100 *μ*g/kg bw/day of triptolide for 35 and 70 days. However, the testicular weights (1.09 ± 0.1 g) of male rats treated with triptolide over a prolonged period (100 *μ*g/Kg bw/day for 82 days) were 26% less than those of the vehicle control (1.48 ± 0.05 g) [17]. Singla et al. [20] also did not observe any significant effect of triptolide treatment (0.025, 0.05, and 0.1% in bait for 7 and 14 days durations) on weights of reproductive organs and accessory sex glands after 30 days of termination of treatment.

**Figure 2:**
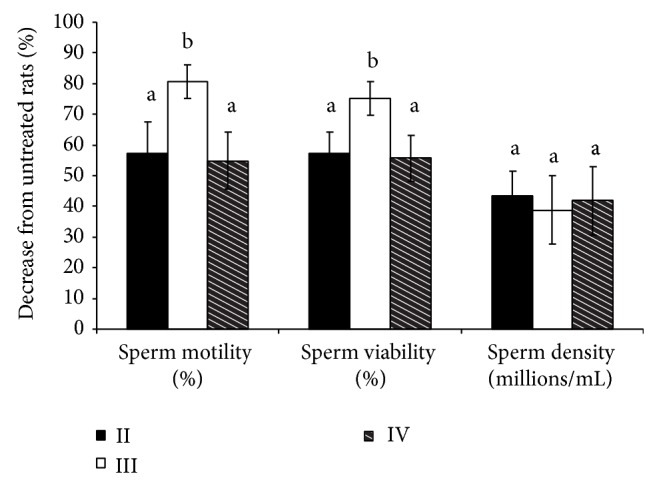
Percent reductions in values of different sperm parameters in groups of rats treated with triptolide from that of untreated group.

A significant (*P* ≤ 0.05) decrease in percent sperm motility and viability and increase in sperm abnormality were found in treated groups of rats compared to untreated group (Table 4). Though the sperm density (millions/mL) was found to be decreased by 38.76 to 43.37% in treated groups of rats from that of untreated group, the differences were not found to be significant statistically. There was no dose dependent effect of triptolide treatment observed on sperm parameters in the cauda epididymal fluid. The highest effect of treatment was observed in rats treated with 0.2% triptolide. The sperm motility (10.00 ± 6.45%) and viability (14.50 ± 7.27%) were found to be reduced significantly (*P* ≤ 0.05) in rats of group III treated with 0.2% triptolide from that of rats of groups II and IV treated with 0.1 and 0.3% triptolide, respectively (Table 4 and Figure 2). Sperm motility and viability which averaged 51.67 and 58.33%, respectively, in rats of untreated group were found decreased by 54.84–80.65% and 55.72–75.14%, respectively, in treated groups of rats (Figure 2).

The sperm motility, which averaged 58.20% in the control rats, was reduced to almost zero in male rats treated orally with 100 *μ*g/kg bw/day of triptolide for 70 days [15]. In rats treated with 100 *μ*g/kg bw/day of triptolide for 82 days also, the sperm motility was reduced to nil by the end of treatment compared with control rats (57.70 ± 0.4%) [17]. Lue et al. [15] observed a decrease in cauda epididymal sperm content by 68% in male rats treated orally with 100 *μ*g/kg bw/day of triptolide for 70 days. In adult male rats fed daily with 100 *μ*g/kg bw of triptolide for 82 days, cauda epididymal sperm content was found decreased by 84% by the end of treatment [17]. The sperm motility and viability were found to range from 5.50 to 34.00 and 13.63 to 39.00%, respectively, in rats of groups treated with 0.025, 0.05, and 0.1% triptolide in bait for 7 days in no-choice feeding test compared to 66.50 and 88.44%, respectively, in untreated group of rats [20]. The poor sperm motility is independent of mitochondrial function as the ATP levels of triptolide treated rats and controls were not found statistically different [17].

**Figure 3:**
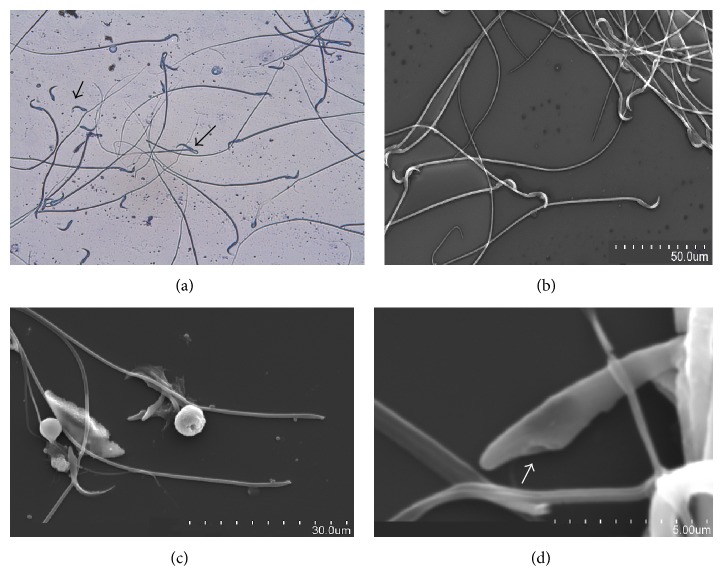
(a–d) Cauda epididymal fluid smear of untreated rats and rats treated with triptolide showing sperm head tail separation. (a) Sperms with head tail separation (short arrow) and midpiece coiling (long arrow) observed in cauda epididymal fluid of treated rat under light microscope at 400x, (b) sperms with no head tail separation observed in cauda epididymal fluid of untreated rat under SEM at 15.0 kV 9.6 mm × 600 SE, (c) sperms with head tail separation observed in cauda epididymal fluid of treated rat under SEM at 15.0 kV 9.9 mm × 1.70 k SE, and (d) sperms with head tail separation observed in cauda epididymal fluid of treated rat under SEM at 15.0 kV 9.9 mm × 10 k SE. Arrow indicates region of separation of middle piece.

The major effect of triptolide treatment on sperm morphology during present studies was sperm head tail separation (Figures 3(a)–3(d)). The SEM of cauda epididymal fluid revealed separation of sperm head at head-midpiece junction. The sperm head tail separation in treated groups of rats ranged from 46.13 to 53.69% (Table 4). The difference in such abnormality was not significant among the three treated groups. Singla et al. [20] observed 36.56 to 51.16% sperm head tail separation in rats treated with 0.025, 0.05, and 0.1% triptolide in bait for 7 days in no-choice feeding test. Triptolide treatment results in nuclear decondensation leading to head tail separation in a severe case and nuclear decondensation without head tail separation in mildly affected cases [17]. Any chromatin decondensation of cauda epididymal sperm nuclei is indicative of sperm malfunction [26] and could also contribute to the observed sterility.

Other abnormalities found in sperm morphology such as abnormal head shape and coiling of midpiece (Figure 3(a)) were found to differ significantly (*P* ≤ 0.05) between rats of treated groups III and IV; however, similar differences with rats of group II were nonsignificant. These abnormalities in treated groups of rats varied from 8.69 to 13.71% (Table 4). Structural abnormalities in epididymal spermatozoa including disrupted connecting pieces, cracked midpieces, and more than 80% of the spermatozoa decapitated in rats treated with 0.05 mg/kg bw/day of tripchlorolide (also obtained from* T. wilfordii*) for 7 weeks were observed by Ye et al. [27]. Virtually all the cauda epididymal sperms in adult Sprague-Dawley rats fed daily with 100 *μ*g/kg bw of triptolide for 82 days exhibited severe structural abnormalities. The most striking changes observed were head tail separation, premature chromatin decondensation of sperm nuclei, a complete absence of the plasma membrane of the entire middle and principal pieces, disorganization of the mitochondrial sheath, and aggregation of many sperm tails [17].

## 4. Conclusion

Feeding of bait containing 0.2% triptolide for 5 days in no-choice feeding test leading to mean daily ingestion of 20.45 mg/kg bw of triptolide was found effective in significantly (*P* ≤ 0.05) reducing sperm motility and viability in cauda epididymal fluid of male* R. rattus *from that of untreated rats. During laboratory breeding also none of the untreated female rats paired with male rats treated with 0.2% triptolide delivered pups. Present studies suggest the potential of 0.2% triptolide bait in regulating reproductive output of* R. rattus.*

## Figures and Tables

**Table 1:** Acceptance of bait containing different concentrations of triptolide fed to male *R. rattus* in laboratory for 5 days in no-choice feeding test.

Groups (*n* = 6 each)	Concentration in bait (%)	Body weight (g)	Acceptance of treated bait over plain bait (%)	Total ingestion of triptolide (mg/kg bw)	Mean daily ingestion of triptolide (mg/kg bw)
I	0.0	152.5 ± 21.9	—	—	—
II∗	0.1	155.0 ± 20.6	93.0 ± 5.7^a^	53.6 ± 7.6^a^	10.8 ± 1.5^a^
III	0.2	145.0 ± 15.0	78.8 ± 2.6^b^	102.2 ± 10.3^b^	20.4 ± 2.1^b^
IV	0.3	185.0 ± 30.8	75.9 ± 16.8^b^	113.0 ± 15.4^b^	22.6 ± 3.1^b^

Values are mean ± SD, *n* = number of rats, and *N* = number of days. ∗One rat died at the end of treatment. Values with different superscripts a–b in a column differ significantly at *P* ≤ 0.05.

**Table 2:** Effect of triptolide treatment on reproductive performance of male *R. rattus* paired with untreated cyclic female rats.

Group (*n* = 3 each)	Conc. in bait (%)	Body weight (g)		Females delivered pups (% pregnancy rate)	Pups delivered/foetuses seen
		Male rats	Female rats		
I	0.0	160.00 ± 23.50	137.60 ± 10.65	3/3 (100%)	7.73 ± 2.05 (5, 7, 10 foetuses)
II	0.1	141.50 ± 18.50	131.50 ± 16.50	1/3 (33.33%)	8 pups
III	0.2	154.00 ± 26.50	137.60 ± 18.50	0/3 (0%)	nil
IV	0.3	175.60 ± 8.65	160.00 ± 7.11	2/3 (66.67%)	5.00 ± 0.00 (5, 5 pups)

Values are mean ± SD; *n* = number of rats.

**Table 3:** Effect of triptolide treatment on weights of reproductive organs and accessory sex glands of male *R. rattus*.

Group (*n* = 6 each)	Conc. in bait (%)	Organ weight (g/100 g bw)			
		Testis	Epididymis	Seminal vesicles	Prostate gland
I	0.0	0.72 ± 0.18^a^	0.51 ± 0.24^a^	0.70 ± 0.24^a^	0.24 ± 0.08^a^
II (*n* = 5)	0.1	0.54 ± 0.10^a^	0.38 ± 0.13^a^	0.62 ± 0.15^a^	0.19 ± 0.06^a^
III	0.2	0.74 ± 0.26^a^	0.55 ± 0.33^a^	0.69 ± 0.33^a^	0.22 ± 0.15^a^
IV	0.3	0.60 ± 0.16^a^	0.39 ± 0.11^a^	0.52 ± 0.32^a^	0.12 ± 0.04^a^

Values are mean ± SD; *n* = number of rats; ^a^mean values in a column sharing common superscript do not differ significantly (*P* ≤ 0.05).

**Table 4:** Effect of triptolide treatment on sperm parameters in cauda epididymal fluid of male *R. rattus*.

Group (*n* = 6 each)	Conc. in bait (%)	Sperm motility (%)	Sperm viability (%)	Sperm density (millions/mL)	Sperm abnormality (%)	
					Head tail separation	Other abnormalities
I	0.0	51.67 ± 7.45^a^	58.33 ± 7.45^a^	148.33 ± 62.23^a^	10.00 ± 4.08^a^	2.58 ± 0.93^a^
II (*n* = 5)	0.1	22.00 ± 11.66^b^	25.00 ± 9.49^b^	84.00 ± 26.34^a^	52.95 ± 18.53^b^	11.42 ± 3.26^b^
III	0.2	10.00 ± 6.45^c^	14.50 ± 7.27^c^	90.83 ± 37.12^a^	46.13 ± 12.15^b^	13.71 ± 4.12^b^
IV	0.3	23.33 ± 10.67^b^	25.83 ± 9.75^b^	130.00 ± 71.82^a^	53.69 ± 24.57^b^	8.69 ± 5.74^b^

Values are mean ± SD; *n* = number of rats; ^a–c^mean values in a column not sharing a common superscript differ significantly (*P* ≤ 0.05).
